# Dispensing and the Insurance Act: The Future of Pharmacy

**Published:** 1913-01-04

**Authors:** 


### DISPENSING AND THE INSURANCE ACT.

### The Future of Pharmacy.

Among the many old customs which the operation of the National Insurance Act threatens to subvert, the exercise by medical men of the privilege of dispensing medicines is by no means the least important. This subversion of the established order of things may not be an unmixed evil even from the point of view of the doctor, while for the pharmacist it undoubtedly opens out prospects of a better future. Apart from the restrictions imposed on the dispensing of medicines by the National Insurance Act there is nothing in English law to prevent medical practitioners from compounding drugs, and since these restrictions only apply to medicines for insurance patients, medical men may continue to dispense medicines for their private patients. There is a possibility, however, that practitioners with a clientèle partly composed of insurance patients and partly of private patients will find it hardly worth while to write prescriptions for one class and supply medicines to the other, and that in course of time they will, like those physicians who in the reign of James I. found dispensing "too servile and laborious a business," delegate this part of their work to the chemists and druggists. The aspect of this question which is likely to affect the art of prescription writing is referred to in our notes this week.

Practitioners in rural districts will be less affected by the new order of things than their colleagues in towns, for the regulations provide that "where an insured person is resident in a rural area at a distance of more than one mile from the place of business of a chemist . . . or where an insured person by reason of distance or inadequacy of means of communication will have difficulty in obtaining any necessary drugs or appliances from a chemist" the practitioner himself may supply the necessary drugs.

Conversely the country chemist will obtain less new business through the Insurance Act scheme than the town chemist. It is roughly estimated that at the present time between 70 and 80 per cent. of the medical practitioners in England and Wales dispense medicines; in Scotland, on the other hand, it is more customary for the doctor to write prescriptions, so that North of the Tweed the change will be less pronounced. Taking Great Britain as a whole, however, it would be correct to assume that for the most part the persons who are now insured have in the past been supplied, when necessary, with medicines by their medical attendants. In future these persons when requiring treatment will, if drugs are ordered, obtain them from chemists.

This will involve a transfer of work from the doctor to the chemist of an extremely important character. The actual sum available for the payment of drugs and appliances will be two shillings multiplied by the number of insured persons—that is to say, something approaching thirty million shillings; the whole of this amount may not be required if the prescribing is on an economical scale, but the probability is that considerably over one million pounds sterling will be divided among the chemists on the panels. Of this a large proportion will be in respect of new business, and to cope with it many pharmacists will find it necessary to add to their staff of assistants, so that there should be little danger of dispensers, who have hitherto been employed by doctors, failing to find employment on terms quite as good as those to which they have been accustomed. On the other hand, the increase in business is not likely to result in a proportionate increase in the chemists' profits; it will, in fact, tend to reduce the percentage of profit, for the prices of medicines supplied to insurance patients will be considerably lower than those charged to ordinary customers; but in the volume of profit there should be a small immediate increase.

The benefit of the Act to pharmacists must not, however, be measured by increased profits so much as by improvement in the character of the business of pharmacy. Hitherto the pharmaceutical side of the chemist's shop has in very many cases been overshadowed by the purely commercial side, for the reason that there has been little occasion for the chemist to exercise the art for which he has been trained, owing to the fact that the dispensing has been done by medical practitioners. In future, therefore, we are likely to witness a change for the better in the status of the chemist and some alteration in the appearance of his shop. An impetus may also be given to pharmaceutical research, for the selling of toilet articles and packed products does not conduce to the scientific frame of mind. In all other European countries the duty of dispensing medicines is confined by law to pharmacists, and pharmacy in those countries is, if not a profitable occupation, a calling which is held in much higher esteem than it is in Great Britain. The English chemist is almost as well equipped by education as his French and German confrère for scientific work; but the nature of the chemist's business on the Continent is conducive to post-graduate study, whereas such is not the case in England. It can at least be hoped that one effect of the operation of the National Insurance Act will be to encourage the English pharmacist to devote more attention to scientific work which leads to improved methods of manufacture and to drug research generally.

